# Exploring transformer models: Fine-tuning VS inference on relation extraction from biomedical texts

**DOI:** 10.1016/j.csbj.2025.12.004

**Published:** 2025-12-20

**Authors:** Hajar El janah, Youness Nachid-Idrissi, Mourad Sarrouti, Said Najah

**Affiliations:** a Laboratory of Intelligent Systems and Applications, Faculty of Sciences and Techniques, Sidi Mohamed Ben Abdellah University, Fez, Morocco; b Sumitovant Biopharma, New York, NY, USA

**Keywords:** Large language models, Pretrained language models, Relation extraction, Named entity recognition, Natural language processing, Generative artificial intelligence

## Abstract

Biomedical data continues to grow significantly, coming from different sources and being updated daily. This makes manual extraction not only time-consuming but also impossible to keep up with due to this constant increase. In this context, biomedical relation extraction, which aims to automate the discovery of relationships between entities from free texts, becomes an essential step for knowledge discovery. While fine-tuning Transformer models such as T5, PubMedBERT, BioBERT, ClinicalT5, and RoBERTa has shown satisfactory results, it requires specific datasets, which are time-consuming to create and costly since they require domain experts. One ideal solution is the use of Generative Artificial Intelligence (GenAI), as it is directly applicable to a problem without the need for data creation. In this paper, we explore these generative large language models (LLMs) to evaluate whether they can be reliable when it comes to processing biomedical data. To do so, we study the relation extraction task of four major biomedical tasks, namely chemical-protein relation extraction, disease-protein relation extraction, drug–drug interaction, and protein–protein interaction. To address this need, our study focuses on comparing the performance of fine-tuned Transformer models with generative models such as Mistral-7B, LLaMA2-7B, GLiNER, LLaMA3-8B, Gemma, RAG, and Me-LLaMA-13B, using the same datasets in both experiments, showing that fine-tuned Transformer models achieve performance levels roughly twice those obtained by generative LLMs. These models require more pretraining on specific data, as demonstrated by Me-LLaMA (pretrained on MIMIC-III), which shows a significant improvement in performance compared to the model pretrained on a general domain. In terms of performance, fine-tuned Transformer models on domain-specific biomedical data achieved average scores ranging from **84.42** to **90.35**, while generative models obtained significantly lower scores, between **36.64** and **53.94**. Among the generative LLMs, LLaMA3-8B, RAG, and Me-LLaMA-13B achieved the top three scores, with Me-LLaMA, pretrained on MIMIC-III, reaching **45.76**, illustrating the benefit of domain-specific pretraining.

## Graphical Abstract

**Figure:**
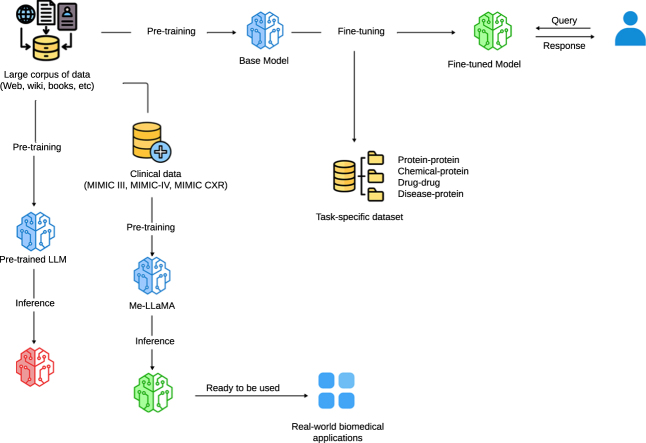

## 1 Introduction

The biomedical literature is growing rapidly, leading to an accumulation of valuable knowledge about proteins, drugs, and diseases. With this rapid growth, manually curating information from the biomedical literature has become increasingly difficult [1]. As a result, Natural Language Processing (NLP) techniques for automatic Relation Extraction (RE) between biomedical entities from texts can provide an interesting way to reduce the time healthcare professionals spend reviewing biomedical literature [2].

In recent years, significant progress has been made in the field of NLP with the advent of large Pretrained Language Models (PLMs), such as BERT [3], RoBERTa [4], GPT-3 [5], BART [6], T5 [7], and others. These models have demonstrated remarkable success in various downstream tasks, leveraging their ability to understand and generate language patterns similar to those of humans. However, creating large training datasets is costly and labor-intensive, particularly in the biomedical domain, as specialized knowledge is required [1].

**Fig. 1:**
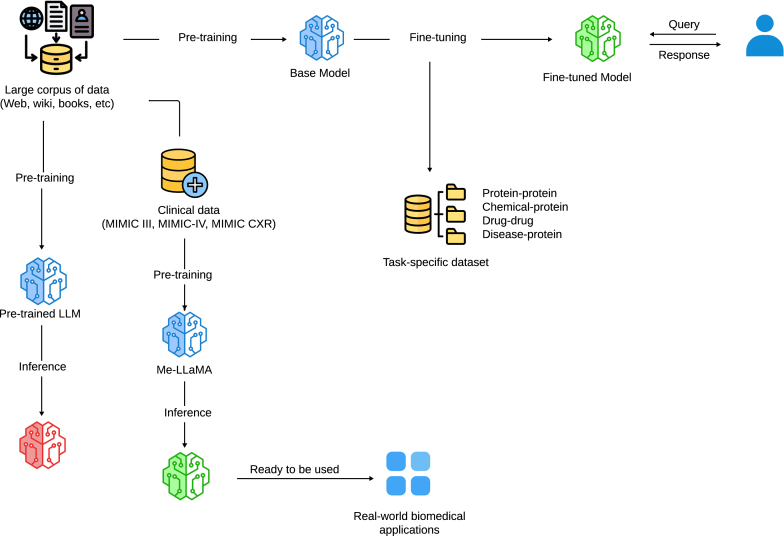
**Architecture of Our Experiment.** This figure illustrates the overall workflow of our experiment. We start with fine-tuning Transformer models on task-specific datasets designed for relation extraction, covering various entity types such as protein-protein, chemical-protein, drug-drug, and disease-protein. After this fine-tuning step, we perform inference using generative LLMs pretrained on large general corpora. Additionally, we use Me-LLaMA, a model pretrained specifically on clinical data, which has demonstrated improved performance compared to models pretrained on general corpora. The process includes both fine-tuning and inference phases, leveraging generative LLMs to assess their suitability for biomedical applications.

To overcome this challenge, our goal is to explore LLMs [8], [9], [10] within the GenAI^1^ approach, without requiring specific or annotated data, to assess whether they can be reliably applied in real-world biomedical applications. To this end, we compare the performance of fine-tuned Transformer models (T5, PubMedBERT, BioBERT, ClinicalT5, and RoBERTa) with that of generative LLMs (Mistral-7B, LLaMA2-7B, GLiNER, LLaMA3-8B, Gemma, RAG, and Me-LLaMA-13B) across four major biomedical tasks: chemical-protein relation extraction [11], [12], disease-protein relation extraction [13], drug–drug interaction [12], [13], and protein–protein interaction. The architecture of our experiment is illustrated in Fig. 1. Our results revealed that fine-tuning Transformer models produces superior performance, with F1-scores reaching up to **90.35**, whereas generative LLMs achieve lower levels. For instance, LLaMA2-7B and Gemma scored **36.64** and **43.29**, respectively, a level far below that of fine-tuned models such as ClinicalT5 (**90.35**) and PubMedBERT (**86.38**). In contrast, Me-LLaMA-13B, pretrained on MIMIC-III, achieved a higher F1-score of **45.76**, outperforming other LLMs (LLaMA2-7B, Gemma, Mistral-7B). This highlights the need for further pretraining on domain-specific data, as demonstrated by Me-LLaMA, which shows a notable improvement in performance.

This paper consists of:

- Fine-tuning small Transformer models on four major biomedical relation extraction tasks: chemical-protein, disease-protein, drug–drug, and protein–protein interactions.
- Performing inference using generative LLMs (e.g., Mistral-7B, LLaMA2-7B, GLiNER, LLaMA3-8B, Gemma, RAG, Me-LLaMA-13B) on the same tasks.
- Analyzing the results of fine-tuning versus inference and demonstrating that Me-LLaMA, pretrained on a specific domain (MIMIC-III), significantly outperforms models pretrained on a general domain.

**Table:** 

Statement of significance	
**Problem or Issue**	Biomedical data are rapidly increasing across multiple sources, making manual extraction inefficient and difficult to scale.
**What is Already Known**	Several studies rely on fine-tuning Transformer models for biomedical relation extraction. However, a major limitation lies in the annotation of specific datasets, which is costly, time-consuming, and requires specialized expertise.
**What this Paper Adds**	Our study explores the use of GenAI models without the need for annotated data, in order to evaluate their reliability in processing real-world biomedical data. We compare the performance of these LLMs with fine-tuned Transformer models, highlighting the need for domain-specific pretraining to improve results.
**Who Would Benefit from the new Knowledge in this Paper**	Medical AI practitioners, clinicians seeking to improve the accuracy and reliability of decision-support systems in real-world biomedical applications, and NLP researchers who can leverage these findings to define future research directions.

## 2 Related works

The NLP community has long been interested in automatically extracting relationships between biomedical entities (such as proteins, genes, and diseases) from biomedical literature [14], [15]. Lately, PLMs have achieved striking success in NLP [16]. Due to this, various transformer-based techniques are extensively used to extract relationships between entities from biomedical literature [11], [17], [18]. However, most pretraining efforts are centered on general-domain corpora, such as news articles and the web. In contrast to the assumption that domain-specific pretraining could benefit from initializing with general-domain language models, study [13] demonstrated that models trained from scratch on biomedical texts achieve significantly better performance than those initialized from general-domain pretraining. Similarly, SciFive [12], which was pretrained exclusively on large biomedical corpora, confirms the effectiveness of domain-specific pretraining.

Recently, large language models (LLMs) have made remarkable progress across multiple NLP tasks, generating growing research interest. In the biomedical domain, several studies illustrate these advances. For example, generative digital twins leverage AI to create realistic synthetic medical data, enabling the simulation of patient scenarios and the training of diagnostic models without compromising privacy [19]. LLMs have also shown promising potential in clinical decision-making, as demonstrated by Gemini Advanced, which achieved 81.87 % accuracy when tested on questions based on real-world cases [20]. In addition, synthetic data generation allows researchers to obtain diverse and statistically representative datasets while preserving patient confidentiality, thereby improving model performance [21] and facilitating integration with data analysis tools within LLMs, enabling optimal utilization of hospital and scientific information for integrated and personalized patient care [22]. Beyond these applications, recent studies have explored strategies to further improve LLM performance and reduce noise in biomedical relation extraction. For instance, Zhang et al. [17] propose a location-enhanced syntactic knowledge approach, which assigns weighted attention to syntactic dependencies, allowing the model to focus on the most relevant parts of a sentence for extracting biomedical relations. Similarly, BiomedRAG [18] enhances LLM outputs by retrieving precise information chunks rather than full sentences, directly integrating them into the model to provide more relevant context and reduce noise in generated responses. Other efforts have focused on leveraging LLMs and Knowledge Graphs (KGs) to enhance biomedical text understanding. BioTextQuest v2.0 [23], for example, provides a tool for clustering and concept discovery in biomedical literature, connecting named entity recognition (NER) with bioinformatics applications. Future versions of BioTextQuest are expected to integrate LLMs to improve literature mining and uncover complex relationships between genes, diseases, and drugs, offering new opportunities for hypothesis generation and discovery. Similarly, BioKGrapher [24] introduces a framework for automated KG construction from PubMed abstracts using NER, entity linking, and ontology alignment. It demonstrates strong concept alignment with medical guidelines and shows promise for enhancing information retrieval and downstream LLM applications. Future directions for BioKGrapher involve combining graph neural networks with retrieval-augmented generation (RAG) to improve biomedical relation extraction and contextual reasoning. Complementary to these text-focused approaches, recent work on multimodal data integration has also emerged. For example, the study in [25] addresses the challenge of classifying and retrieving medical images within data integration centers using a deep learning model (ResNet50). By generating standardized annotations aligned with terminologies such as SNOMED CT, this approach contributes to improving the semantic interoperability of medical data, which could further benefit text-based retrieval systems and LLM-driven biomedical knowledge extraction.

Building on these insights, our study investigates how LLMs perform on biomedical relation extraction without domain-specific pretraining. In this work, we perform comprehensive comparisons between Transformer models (fine-tuned on domain-specific data) and generative LLMs (pretrained on general domain corpora) across multiple biomedical relation extraction datasets, examining their performance in the biomedical domain without domain-specific pretraining.

## 3 Experiments and methods

### 3.1 Datasets

We investigated six benchmark datasets designed for RE, covering diverse entity types such as protein–protein, drug–drug, chemical-protein, and disease-protein. The details of the train, dev, and test splits for the biomedical RE datasets are provided in Table 1.

**Table 1:** Statistics of the biomedical RE datasets. For GAD, AIMed and BioInfer, we use the dev set as a test set.

Dataset	Train	Dev	Test	Metrics
AIMed	4938	549	549	micro F1
BioInfer	8544	950	950	micro F1
DDI	2937	1004	979	micro F1
ChemProt	4154	2416	3458	micro F1
DrugProt	17,281	3763	3765	micro F1
GAD	4796	534	534	micro F1

**Protein–protein interactions:** When focusing on protein–protein interactions, we utilized two benchmark datasets, specifically BioInfer and AIMed. To maintain anonymity, we used the predetermined tag @PROTEIN$ to mask the identities of the entities mentioned in a sentence. For example, a sentence containing two protein names is transformed as follows: “Analysis of @PROTEIN$, p53, p16, and @PROTEIN$/Smad4”.

**Drug–drug interactions:** We employed a preprocessed edition of the Drug–Drug Interaction (DDI) 2013 corpus as provided in [2]. Drug names were anonymized using the tag @DRUG$. For example, a sentence containing a pair of drug names is presented as “The effect of @DRUG$ on oral @DRUG$ is variable”. We assess four types of Drug–Drug Interaction (DDI) relationships: “mechanism”, “effect”, “advise” and “int”. The “mechanism” category refers to DDIs described by their pharmacokinetic (PK) mechanism. The “effect” type is used to annotate DDIs describing an effect. The “advise” class is employed when a recommendation or advise regarding a drug interaction is provided. The “int” class is utilized when a DDI is mentioned in the text without providing any additional information.

**Disease-protein relationships:** We used the preprocessed editions of the Genetic Association Database corpus (GAD) as introduced in [26]. Entities of interest were anonymized with the tags @DISEASE$ and @GENE$. For example, a sentence with a pair of these entities is expressed as “MPO genotype GG is associated with @DISEASE$ in patients with hereditary @GENE$”.

**Chemical-protein relationships:** We employed the datasets ChemProt [14] and DrugProt [15], which encompass gene–chemical relations. We assess five classes, summarized in Table 2, which lists the relation labels and their corresponding subgroups for ChemProt. For DrugProt, we used the standard training and development sets from the DrugProt shared task and assessed 13 classes: Activator, Agonist, Agonist-Inhibitor, Antagonist, Direct-Regulator, Indirect-Downregulator, Indirect-Upregulator, Inhibitor, Part-Of, Product-Of, Substrate, Substrate-Product-Of, Agonist-Activator (see the detailed guidelines for chemical–protein relations in [27]).

**Table 2:** ChemProt relation labels and their associated subgroups [27].

Label	Subgroups
CPR:3	upregulator, activator, indirect upregulator
CPR:4	downregulator, inhibitor, indirect downregulator
CPR:5	agonist, agonist-activator, agonist-inhibitor
CPR:6	antagonist
CPR:9	substrate, product of

### 3.2 Transformer models

**Transformers** are a class of deep learning models that leverage self-attention mechanisms to assign varying importance to different components of the input. Since their introduction by Vaswani et al. [28] in 2017, they have become widely used in areas such as NLP and computer vision. The original transformer architecture defines two main parts, an encoder and a decoder.^2^ One well-known example of a transformer-based encoder-decoder model is T5 (Text-to-Text Transfer Transformer), proposed by Raffel et al. [7] and released by Google in 2019. T5 is highly versatile and can be adapted to numerous NLP tasks, including translation, question answering, relation extraction, and many others. Another influential transformer model is BERT (Bidirectional Encoder Representations from Transformers), introduced by Devlin et al. [3] and launched by Google in 2018. BERT is initially trained on extensive textual data and can later be fine-tuned to perform a variety of NLP applications.^3^

### 3.3 BERT and T5 pretraining strategy and model architectures

In this comparative study, we evaluate three BERT variants—BioBERT, RoBERTa, and PubMedBERT—and one T5 variant (encoder-decoder architecture), ClinicalT5, for extracting relationships across the datasets described in Section 3.1. Specific details for each model are provided in the appendices.

#### 3.3.1 Models: architectures

**BioBERT**, is a domain-specific extension of the BERT architecture, tailored for natural language processing tasks in the biomedical field. For relation extraction, it adopts the sentence classification mechanism from the original BERT model, where a [CLS] token is employed to identify the relationship between entities. The classification relies on a single output layer built upon the [CLS] token’s representation. To standardize input and protect entity identity, named entities within sentences are replaced by predefined tags such as @GENE$ and @DISEASE$. For instance, a sentence involving a gene and a disease might appear as: “Serine at position 986 of @GENE$ may be an independent genetic predictor of angiographic @DISEASE$.” [29]. **PubMedBERT**, is a biomedical language model pretrained from scratch on PubMed abstracts and PubMed Central full-text articles, built upon the original BERT architecture [13].^4^

**The RoBERTa**^5^ was introduced as an improved version of BERT [4]. Developed from Google’s original BERT model (2018), RoBERTa includes several enhancements, such as adjustments to key hyperparameters. Notably, it removes the next-sentence prediction task and is trained with larger mini-batches and higher learning rates, improving performance across various NLP benchmarks. The **T5** (Text-To-Text Transfer Transformer) architecture is a transformer-based language model developed by Google [7]. Its key principle is to treat all NLP tasks as text-to-text tasks, meaning both the input and output are formulated as textual sequences.

#### 3.3.2 Models: pretraining

**BioBERT**, as a specialized language representation model for biomedical text, underwent pretraining on datasets distinct from its general-purpose counterpart, BERT. While BERT was pretrained on English Wikipedia and BooksCorpus, BioBERT’s pretraining utilized PubMed abstracts and PubMed Central full-text articles (PMC). Table 3 provides a detailed listing of the text corpora used for BioBERT pretraining [29].

**Table 3:** Summary of corpora used in BioBERT, based on [29].

Corpus	Number of words	Domain
English Wikipedia	2.5B	General
BooksCorpus	0.8B	General
PubMed Abstracts	4.5B	Biomedical
PMC Full-text articles	13.5B	Biomedical

**ClinicalT5** [30], following prior studies on clinical language models [31], [32], is trained on textual notes from MIMIC-III, comprising approximately 2 million notes. Minimal preprocessing is applied, removing unnecessary tokens and characters [31]. The model weights are initialized from the SciFive PubMed-PMC model (base and large) [33] and further pretrained using the span-mask denoising objective [7] on the preprocessed MIMIC-III notes.

### 3.4 Inference

Inference with generative LLMs offers an efficient solution without technical constraints. It allows users to directly leverage the capabilities of pretrained models without additional training or fine-tuning, facilitating the rapid addressing of real-world problems. This represents a significant advantage in clinical environments, where decisions often need to be made quickly to improve patient care. Moreover, the inference approach also reduces costs, as it does not require retraining models on domain-specific data, unlike fine-tuning. Instead, it simply involves providing a prompt to guide the model in performing the desired task. This allows users to access models with minimal effort while staying up to date with the latest versions of pretrained models.

#### 3.4.1 Models: architectures

**Me-LLaMA-13B-chat** [34]: To achieve inference on biomedical tasks, we used the Me-LLaMA-13B model on several datasets, including AIMed, ChemProt, DDI, and GAD. The Me-LLaMA-13B model was continually pretrained from the LLaMA2-13B model, incorporating data from biomedical (PubMed Central and PubMed Abstracts sourced from the Pile dataset), clinical (MIMIC III, MIMIC-IV, and MIMIC-CXR), and general domains in a 15:1:4 ratio. This combination enabled the model to develop strong capabilities in processing medical texts while maintaining a broad general knowledge base.

**Fig. 2:**
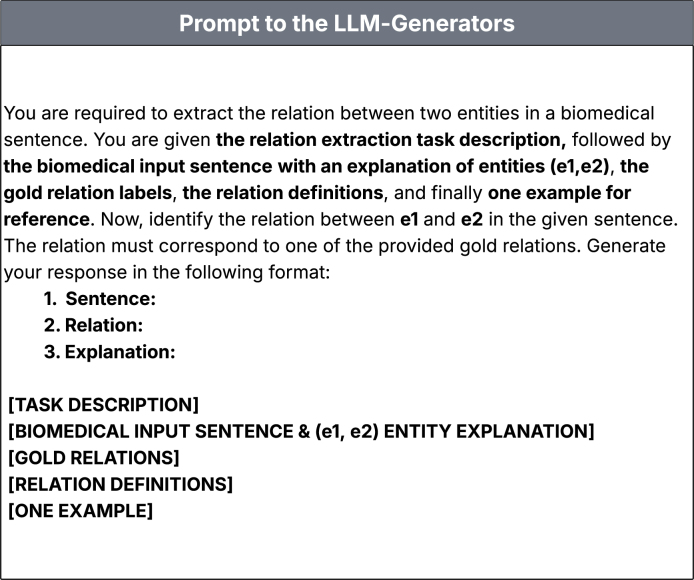
Prompt template used for the LLM-Generators.

**Retrieval Augmented Generation (RAG)** [35]: RAG is a widely adopted method that complements the capabilities of LLMs by providing access to external information, particularly for content not included in their original training data. While LLMs can generalize and infer patterns from their training, they may still produce inaccurate or outdated responses when asked about information that is missing or evolving. Fine-tuning an LLM on new data can address this issue but is often costly and time-consuming. RAG offers an alternative by dynamically retrieving relevant contextual information from external sources, allowing the model to generate more accurate and up-to-date outputs. In our experiments, we used:

• **LLM**: Zephyr-7B-β, a fine-tuned variant of mistralai/Mistral-7B-v0.1, trained on a combination of public and synthetic datasets using Direct Preference Optimization (DPO)^6^ [36].
• **Documents**: PubMed Articles on ‘Cancer’.
• **RAG Implementation**: In our implementation, the RAG pipeline follows a retrieval–generation workflow. The knowledge base was built from PubMed abstracts, segmented into 512-character chunks with 30-character overlap using a recursive text splitter. We used the BAAI/bge-base-en-v1.5 model to generate dense embeddings, stored in a FAISS vector database for efficient similarity search. For each query or sentence, the retriever selects the top four most relevant chunks, which are passed as context to the Zephyr-7B-β model. The model then generates the final output using a structured prompt designed for relation extraction (Fig. 2, showing the prompt template used for the LLM-Generators).

**GLiNER** [37]: A NER model that can identify various entity types using a bidirectional transformer encoder (BERT-like). It provides a flexible and efficient solution for recognizing entities beyond predefined categories, making it suitable for diverse domains and practical applications.

**Gemma** [38]: A family of lightweight text-to-text language models from Google, available in English, with pretrained and instruction-tuned variants. These models can perform tasks such as question answering, summarization, and reasoning, and can run efficiently on devices with limited resources.

**Mistral-7B** [36]: A 7-billion parameter language model based on a transformer architecture [28]. Released in late 2023, it is trained with a dense architecture and achieves superior results on standard NLP benchmarks compared to many larger models.

**LLaMA2-7B** [39]: A family of LLMs developed by Meta, ranging from 7B to 70B parameters. The models are optimized for dialogue tasks using supervised fine-tuning (SFT) and reinforcement learning with human feedback (RLHF). **LLaMA3-8B** [40], the next generation of Meta’s language models, available in 8B and 70B sizes. It uses an optimized transformer architecture and applies SFT and RLHF to enhance performance, helpfulness, and safety in dialogue applications.

#### 3.4.2 Prompting and LLM outputs

**Fig. 3:**
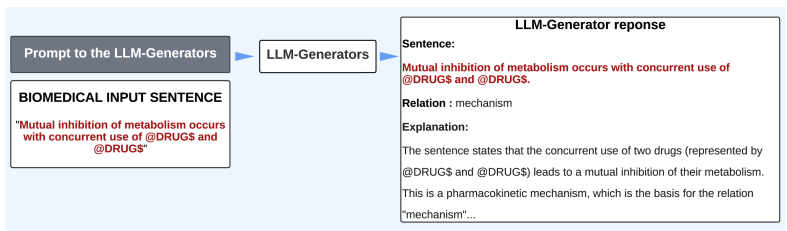
**LLM-generated output for DDI (drug–drug interaction) relation extraction.** The biomedical input sentence, predicted relation, and explanation are shown to illustrate how the model identifies the relationship between the entities based on the provided prompt.

To utilize generative LLMs in relation extraction, we first design the prompt. Each prompt includes the task description, the biomedical input sentence with explanations of the entities $e_{1}$ and $e_{2}$, the gold relation labels, relation definitions, and one illustrative example (Fig. 2). The model is instructed to generate a structured output consisting of the sentence, the predicted relation, and an explanation (Fig. 3, showing the LLM-generated output for DDI relation extraction).

During inference, the prompt is sent to the model $f_{\theta }$ to predict the relation label. If the model does not return a label, the prompt is re-sent to regenerate the response. Subsequently, post-processing is applied to extract the predicted label $l_{i}$ for each sentence $s_{i}$, which is then stored along with the corresponding input sentence (Algorithm 1, outlining the one-shot prompting process for relation extraction, including prompt construction, inference, and post-processing steps).

Algorithm 1 **One-shot Prompting for Relation Extraction**

### 3.5 Experimental setups

In our experiments, we used T5-base, ClinicalT5-base, BioBERT, PubMedBERT (BiomedNLP-BiomedBERT-base), and RoBERTa implementations, all provided via Hugging Face’s Transformers library [41]. Each model was fine-tuned on the respective datasets (using the same train, dev, and test splits) for our relation extraction tasks. All models were trained with a batch size of 8 and maximum sequence length of 300 tokens for 8 epochs using single GPU (15GB VRAM) on Google Colab. Adam optimizer with a learning rate of 1e-5 was used. For inference with generative LLMs, we used a temperature value of 0.7, with other decoding parameters set to the default values provided by HuggingFacecitehuggingface2019 for the open-source models. LLMs were evaluated exclusively on the test splits, ensuring that no training or validation data was used during the generation process. We selected a temperature value of 0.7 to balance coherence and diversity in LLM-generated responses, ensuring a robust evaluation across various output scenarios. Since the generative LLMs do not need to produce extremely long outputs, the maximum output tokens were set to 300 tokens for most models, except for Me-LLaMA-13B, where the maximum was set to 1500 to handle longer prompts and more complex responses, and for RAG, where the maximum tokens were limited to 300–700 to optimize memory usage and ensure efficient retrieval-augmented generation. All generative LLM experiments were executed using A100 GPUs on Google Colab Pro. For small models such as T5 and GLiNER, outputs are deterministic, whereas for LLMs, we performed multiple runs (10 runs).

### 3.6 Results

In Table 4, we present the results of Transformer models, including T5, pretrained on a mixture of tasks (e.g., question answering, named entity recognition, reasoning, and text classification), as described in the original article [42] and shown in Fig. 2 of that article; PubMedBERT, pretrained on PubMed abstracts and PubMed Central; ClinicalT5, pretrained on MIMIC-III medical records; RoBERTa, fine-tuned on the Language Identification dataset; and BioBERT, pretrained on PubMed abstracts and full-text PubMed Central (PMC) articles, on six benchmark datasets for biomedical RE, listed in Table 1. ClinicalT5, pretrained on MIMIC-III notes, achieved a higher F1 score (**90.35**) than T5 (**89.85**), RoBERTa (**88.44**), PubMedBERT (**86.38**) and BioBERT (**84.42**). ClinicalT5 achieved the highest F1 scores on three of the six biomedical RE datasets: ChemProt (**93.37**), AIMed (**92.90**) and DrugProt(**89.74**). T5, trained on a general domain corpus, achieved a higher F1 score (**89.85**) than BioBERT (**84.42**), PubMedBERT (**86.38**) and RoBERTa (**88.44**). T5 obtained the highest F1 scores on one dataset (GAD) and showed competitive results on other datasets. T5 significantly outperformed PubMedBERT, highlighting the value of text generation models. This result demonstrates that text-to-text (text generation) models are highly versatile and broadly applicable within domain-specific contexts, explaining why T5, even as a general-domain model, can surpass BioBERT. RoBERTa showed strong performance on BioInfer (**93.68**) and DDI (**92.83**), despite being trained on a different corpus.

**Table 4:** Biomedical relation extraction test results. Models were fine-tuned on six datasets and evaluated on their corresponding validation sets.

Relation	Dataset	BioBERT	PubMedBERT	T5	ClinicalT5	RoBERTa
Protein–protein	AIMed	86.52	86.89	91.80	**92.90**	90.34
	BioInfer	81.16	84.42	93.05	93.58	**93.68**
Chemical–protein	ChemProt	90.93	93.24	92.75	**93.37**	90.94
	DrugProt	83.98	85.07	88.41	**89.74**	86.45
Drug–drug	DDI	92.39	91.91	92.73	92.30	**92.83**
Disease–protein	GAD	71.54	76.78	**80.34**	80.23	76.44
Average score		84.42	86.38	89.85	**90.35**	88.44

The following results Table 5 concern generative LLMs evaluated in inference mode for biomedical relation extraction, comparing their performance on four datasets representing specific relations: AIMed (protein–protein), ChemProt (chemical-protein), DDI (drug–drug), and GAD (disease-protein), along with 95 % confidence intervals. The LLaMA3-8B model achieved the highest F1 scores on two of the four biomedical RE datasets, with an F1 score of **76.64** on ChemProt (which outperforms the fine-tuning of LLaMA3 reported in [18], Table 6). The Me-LLaMA model outperforms Mistral, LLaMA2, and Gemma with a higher F1 score of **45.76**. For the protein–protein relation, it even surpasses these models along with LLaMA3 (**19.67**), thereby doubling the score (**38.23**). It also outperforms Mistral, LLaMA2, and GLiNER, for chemical-protein relations. In the case of disease-protein relations, it outperforms RAG. Finally, GLiNER, designed for NER, does not provide results on binary datasets (AIMed and GAD), which is consistent with its specialization.

**Table 5:** Biomedical relation extraction test results. Inference models evaluated on four datasets (micro F1-scores with 95 % confidence intervals).

*Notes*: GLiNER is deterministic; for LLMs, results were averaged over 10 runs. The green color represents the values of the Me-LLaMA model, while the red color indicates all values below those of Me-LLaMA. This coloring is used to highlight and emphasize the performance of the Me-LLaMA model compared to the other models.

**Table 6:** Related Works: Transformers vs Generative LLMs Overview.

*Notes*: Comparison of model performance on the ChemProt, DDI, and GAD datasets. The Transformer models were fine-tuned using the same data splits (train, dev, test) for these datasets. LLaMA3 and Me-LLaMA are fine-tuned variants, whereas our approach relies on inference using these models. F1 scores correspond to micro-F1 values taken from the literature using the same data splits. Our reported scores are highlighted in bold blue. The best scores are indicated in the last row of the table.

These results highlight the performance gaps between fine-tuned models and generative LLMs. The differences in performance can primarily be attributed to several key factors:

**Data Coverage**: Transformer models benefit from comprehensive coverage of task-specific data, as seen in AIMed and ChemProt, where relationships between proteins are well-covered. However, generative LLMs, which rely on inference and external context retrieval (RAG), do not possess the same richness of task-specific data and may not capture all the nuances of biomedical relationships.

**Lack of Domain Pretraining**: A model fine-tuned on a biomedical corpus is exposed to the specificities of the domain, including specialized terms, sentence structures, and domain-specific relationships. Generative LLMs in inference mode do not have the same prior exposure to biomedical data, resulting in lower performance compared to Transformer models.

**Task Structure (Binary vs. Multi-class)**: A slight drop in performance is observed on certain datasets, such as AIMed. The difficulty of the task or dataset can impact overall performance. Specifically, in AIMed, where the entities are similar within the same sentence (e.g., “Analysis of @PROTEIN$, p53, p16 and @PROTEIN$/Smad4”), many LLMs perform poorly, with results below 30 %. This suggests that LLMs struggle to distinguish between two instances of the same entity (@PROTEIN$) in the same phrase. On the other hand, for multi-class datasets (DDI and ChemProt) and datasets like GAD, involving different entities (e.g., “MPO genotype GG is associated with @DISEASE$ in patients with hereditary @GENE$”), most LLMs perform above 50 %, indicating they can better handle the distinction between multiple relation classes, as well as between @DISEASE$ and @GENE$. Transformer models are better suited to handle the binary task structure, and fine-tuning helps them learn subtle distinctions even in complex multi-class tasks. Generative LLMs may not be as well-prepared for these distinctions.

LLMs have shown lower performance compared to fine-tuned Transformer models. For these LLMs to be ready for use in real-time biomedical applications, they require more pretraining on domain-specific data, as demonstrated by Me-LLaMA (pretrained on MIMIC-III) and ClinicalT5 (trained on MIMIC-III), which show significant improvement compared to models pretrained on a general domain. In addition to the need for domain-specific pretraining, in-context learning (ICL) could offer a promising approach to narrow the performance gap between inference-only LLMs and fine-tuned models, particularly in biomedical tasks. ICL has emerged as a favored approach for addressing a wide array of tasks without the need for extensive fine-tuning. It allows LLMs to learn from examples without changing their weights, and could be especially useful for long-context models that can leverage multiple examples. Including an ablation or prompt-sensitivity analysis in future work would help clarify the extent to which ICL contributes to performance improvements and provide more visibility into its effectiveness.

### 3.7 Error analysis

#### 3.7.1 Fine-tuning: T5

We conducted a manual review of the test sets to analyze instances where T5 (pretrained on a mixture of tasks), the second-best performing model after ClinicalT5, produced incorrect label predictions. Table 7 provides illustrative examples.

**Table 7:** EA - The misclassified sentence by the T5 model.

In the table, the gold label is shown in red, the predicted label is shown in green, and the keywords used in ErrorAnalysis are highlighted in blue. This coloring is used to clearly distinguish between the reference labels, model predictions, and important keywords for analysis.

**Protein–protein relationships:** Sentences containing “and” were frequently misclassified (examples **1** and **3**). In example **1**, 52.6 % of the errors were linked to syntactic ambiguity caused by the conjunction “and”. This is because the preposition “and” can be confusing for the model, which may interpret the sentence as describing two separate events or subjects (Table 8, shows the frequency of error types across the datasets using the T5 model). Additionally, the presence of parentheses and expressions within parentheses, such as (“PROTEIN$ SC1”), can add noise and be interpreted by the model as a specification or particular characteristic of the protein mentioned. Consequently, the model might consider that @PROTEIN$ SC1 has a direct relationship with @PROTEIN$ (example **2**, 71.6 %, Table 8).

**Table 8:** Frequency of error types across ChemProt, DDI, and AIMed datasets using T5 model, including the percentage of errors associated with ambiguous terms, the specific terms contributing to each error, and the corresponding error taxonomy.

Dataset	Error type	% Errors with ambiguous terms	% per Term	Error taxonomy
ChemProt	CPR3 → CPR5	57.2 %	promote 0.8 %, increase 23.8 %, activate 13.5 %, activator 5.6 %, stimulate 4.0 %, reduce 9.5 %	**Lexical Overlap:** caused by classes sharing the same words (e.g., CPR3 and CPR5).
ChemProt	CPR4 → CPR6	69.6 %	inhibitors 69.6 %	**Lexical Overlap:** similar inhibitory terms shared between classes.
ChemProt	CPR9 → CPR6	**31.2** %	inhibitor 17.0 %, inhibited 1.9 %, reduction 1.9 %, reduces 1.9 %, are metabolized 8.5 %	**Lexical Overlap** for shared inhibitory terms.
ChemProt	CPR4 → CPR9	30.0 %	inhibition 30.0 %	**Lexical Overlap**.
ChemProt	CPR4 → CPR5	47.2 %	decrease 6.5 %, inhibitor 40.7 %	**Lexical Overlap:** overlapping inhibitory and decrease-related words between classes.
DDI	Mechanism → Effect	50.9 %	Mixing 11.3 %, inhibit 18.9 %, metabolism 9.4 %, absorption 11.3 %	**Semantic Ambiguity: Action vs Result** — terms like “Mixing”,“inhibit” and “metabolism” were often confused as effects rather than mechanisms;
				**Multi-Entity Complexity:** terms like “absorption”, created confusion by being used in both contexts of mechanism and effect.
DDI	Advise → Mechanism	**39.0** %	inhibition 12.2 %, combination 14.6 %, interactions 12.2 %	**Semantic Ambiguity: Action vs advise**.
AIMed	True → False	52.6 %	“and” 52.6 %	**Syntactic Ambiguity:** confusion caused by sentence structure or conjunctions.
AIMed	False → True	71.6 %	(parentheses) 71.6 %	**Syntactic Ambiguity:** ambiguous parenthetical phrases.

*Notes*: The arrow (→) indicates misclassification, i.e., the label on the left was misclassified as the label on the right. For example, CPR3 → CPR5 means that CPR3 instances were misclassified as CPR5.

**Chemical-protein relationships:** In the context of CPR classification, the CPR:3 class is often predicted as CPR:5 (example **6**). The CPR:3 class usually describes up-regulation, with instances containing up-regulation words such as “promote” (0.8 %), “increase” (23.8 %), and “activate” (13.5 %). The CPR:5 class typically relates to receptor modulation and contains activation or inhibition words such as “activate”, “increase”, “stimulate” (4.0 %), and “reduce” (9.5 %). The presence of the word “activate” in both CPR:3 and CPR:5 can lead to misclassification, as observed in 13.5 % of the cases with T5 (Table 8). The CPR:4 class generally refers to down-regulation and includes words such as “decrease” (6.5 %), “inhibitor” (40.7 %), and “deposition”. The presence of the word “inhibitors” in both CPR:4 (INHIBITOR) and CPR:5 (AGONIST-INHIBITOR) creates confusion for the model (example **10**). The verb “inhibit” describes the action of pharmacological agents. Since CPR:6 corresponds to ANTAGONISTS that reduce the action of @CHEMICAL$, the model could misinterpret the term “inhibitors” (69.6 %) mentioned in the sentence (example **7**). Similarly, the model may have misinterpreted the verb “are metabolized” (8.5 %) and associated it with an antagonistic action rather than a metabolic process, due to its similarity with other terms used in antagonistic contexts (example **8**). The model also misclassified the CPR:4 and CPR:9 classes because of the term “inhibition” (30.0 %), shared between CPR:4 (INHIBITOR) and CPR:9 (SUBSTRATE in the context of chemical interaction) (example **9**).

**Drug–drug relationships:** The presence of the verb “Mixing” (11.3 %) twice in a sentence could contribute to model confusion. The verb indicates an action of merging two substances, which the model interpreted as an effect rather than a mechanism (example **4**). Additionally, the expression “potential interactions” (12.2 %) may lead to errors, as it suggests the possibility of interactions between the drugs mentioned. The model could therefore misinterpret the context of the sentence and incorrectly predict that the relationship between the drugs is a mechanism rather than advise (example **5**).

#### 3.7.2 Inference: GLiNER, mistral and LLaMA3

To analyze the inference behavior of our models, we conducted an error analysis for GLiNER, Mistral, and LLaMA3, the best-performing models. Our manual review highlights patterns of misclassification that reveal the challenges each model faces in relation extraction tasks.

• **GLiNER:**
  **DDI [*int*** → **effect]**
  The confusion between these two types may result from the difficulty in distinguishing between a mention of interaction that describes a specific effect and a mention that only provides information about the existence of the interaction without detailing its effect or mechanism (**Table A.1** presents the confusion matrix).
  **ChemProt** [***CPR3*** → **CPR9**] [***CPR4*** → **CPR9**]
  The confusion between CPR3 and CPR9, as well as CPR4 and CPR9, may arise from the difficulty in distinguishing interactions that directly regulate the activity of proteins or genes (CPR3 and CPR4) from those that simply involve the chemical compound as a substrate or product of an enzymatic reaction (CPR9) (**Table A.2**).
• **Mistral:**
  **ChemProt** [***CPR3*** → **CPR4**]
  The confusion between CPR3 and CPR4 may result from a difficulty in distinguishing interactions that upregulate (CPR3) from those that downregulate (CPR4) the activity of proteins or genes (**Table A.3**).
  **DDI** [***effect*** → **mechanism**] [***int*** → **mechanism**]
  The confusion where “effect” is predicted as “mechanism” and “int” is also predicted as “mechanism” may stem from overlap in the descriptions of these types in the annotation guidelines (**Table A.4**).
  For example, “effect”, encompasses descriptions of both specific effects and pharmacodynamic mechanisms. Similarly, “int” refers to interactions mentioned in the text without additional details. When these descriptions are not sufficiently distinct, or when ambiguity exists in the text, the model may misclassify them as “mechanism”, which is used for annotations specifically related to pharmacokinetic mechanisms.
• **LLaMA3:**
  Predictions for both ChemProt (**Table A.5**) and DDI (**Table A.6**) were largely accurate, with minimal errors. However, in the binary GAD dataset (**Table A.7**), a significant issue was observed: “False” labels were often predicted as “True”, indicating that the model incorrectly identified a relationship between @DISEASE$ and @GENE$ entities when none actually existed.
  ***Note***: Tables **A.1** to **A.7** of the Supplementary Materials present the confusion matrices.

#### 3.7.3 Error taxonomy: T5, Me-LLaMA and LLaMA3

To better understand the errors made by our models, we categorized the errors into four main classes based on their nature: Lexical Overlap, Semantic Ambiguity, Multi-Entity Complexity and Syntactic Ambiguity. This categorization allowed us to analyze the performance of the T5, Me-LLaMA, and LLaMA3 models on the ChemProt, DDI, and AIMed datasets in greater depth. We used the following tables to support our analysis: Table 8 presents the frequency of error types across the ChemProt, DDI, and AIMed datasets using the T5 model, including the percentage of errors associated with ambiguous terms and their corresponding error taxonomy. These results are discussed in Section 3.7.1. Table 9, Table 10 present the frequency of errors for LLaMA3 and Me-LLaMA, respectively.

**Table 9:** Frequency of error types across ChemProt, DDI and AIMed datasets using LLaMA3 model, including the percentage of errors associated with ambiguous terms, the specific terms contributing to each error, and the corresponding error taxonomy.

Dataset	Error type	% Errors with ambiguous terms	% per Term	Error taxonomy
ChemProt	CPR3 → CPR5	**42.5** %	promote 0 %, increase 12.5 %, activate 22.5 %, activator 2.5 %, stimulate 5 %, reduce 0 %	**Lexical Overlap:** caused by classes sharing the same words (e.g., CPR3 and CPR5).
ChemProt	CPR4 → CPR6	**28.89** %	inhibitors 28.89 %	**Lexical Overlap:** similar inhibitory terms shared between classes.
ChemProt	CPR9 → CPR6	40 %	inhibitor 20 %, inhibited 20 %, reduction 0 %, reduces 0 %, are metabolized 0 %	**Lexical Overlap** for shared inhibitory terms(“inhibitor”, “inhibited”)
ChemProt	CPR4 → CPR9	33.33 %	inhibition 33.33 %	**Lexical Overlap**
ChemProt	CPR4 → CPR5	42.42 %	decrease 0 %, inhibitor 42.42 %	**Lexical Overlap** confusion due to shared inhibitory term (“inhibitor”)
DDI	Mechanism → Effect	**25.3** %	Mixing 0 %, inhibit 11.7 %, metabolism 0 %, absorption 13.6 %	**Semantic Ambiguity: Action vs Result** — term like “inhibit” was often confused as effects rather than mechanisms;
				**Multi-Entity Complexity:** terms like “absorption”, created confusion by being used in both contexts of mechanism and effect.
DDI	Advise → Mechanism	60 %	inhibit 20 %, combination 0 %, interactions 40 %	**Semantic Ambiguity: Action vs advise**.
AIMed	True → False	**0** %	“and” 0 %	**Syntactic Ambiguity:** None
AIMed	False → True	**50.34** %	(parentheses) 50.34 %	**Syntactic Ambiguity:** ambiguous parenthetical phrases.

*Notes*: The arrow (→) indicates misclassification, i.e., the label on the left was misclassified as the label on the right. For example, CPR3 → CPR5 means that CPR3 instances were misclassified as CPR5.

**Table 10:** Frequency of error types across ChemProt, DDI, and AIMed datasets using Me-LLaMA model, including the percentage of errors associated with ambiguous terms, the specific terms contributing to each error, and the corresponding error taxonomy.

Dataset	Error type	% Errors with ambiguous terms	% per Term	Error taxonomy
ChemProt	CPR3 → CPR5	43.75 %	promote 0 %, increase 12.5 %, activate 6.25 %, activator 6.25 %, stimulate 12.5 %, reduce 6.25 %	**Lexical Overlap:** caused by classes sharing the same words (e.g., CPR3 and CPR5).
ChemProt	CPR4 → CPR6	57.14 %	inhibitors 57.14 %	**Lexical Overlap:** similar inhibitory terms shared between classes.
ChemProt	CPR9 → CPR6	33.33 % (<% LLaMA3)	inhibitor 0 %, inhibited 33.33 %, reduction 0 %, reduces 0 %, are metabolized 0 %	**Lexical Overlap** for shared inhibitory terms(“inhibited”)
ChemProt	CPR4 → CPR9	**9.52** % (<% LLaMA3)	inhibition 9.52 %	**Lexical Overlap**
ChemProt	CPR4 → CPR5	**20.69** % (<% LLaMA3)	decrease 0 %, inhibitor 20.69 %	**Lexical Overlap** confusion due to shared inhibitory term (“inhibitor”)
DDI	Mechanism → Effect	31.9 %	Mixing 0 %, inhibit 17.3 %, metabolism 0 %, absorption 14.6 %	**Semantic Ambiguity: Action vs Result** — term like “inhibit” was often confused as effects rather than mechanisms;
				**Multi-Entity Complexity:** terms like “absorption”, created confusion by being used in both contexts of mechanism and effect.
DDI	Advise → Mechanism	56 % (<% LLaMA3)	inhibit 32.9 %, combination 2.4 %, interactions 20.7 %	**Semantic Ambiguity: Action vs advise**.
AIMed	True → False	20 %	“and” 20 %	**Syntactic Ambiguity:** confusion caused by sentence structure or conjunctions.
AIMed	False → True	53.16 %	(parentheses) 53.16 %	**Syntactic Ambiguity:** ambiguous parenthetical phrases.

*Notes*: The arrow (→) indicates misclassification, i.e., the label on the left was misclassified as the label on the right. For example, CPR3 → CPR5 means that CPR3 instances were misclassified as CPR5.

After analyzing the performance of the LLaMA3 and Me-LLaMA models on the ChemProt, DDI, and AIMed datasets, we observed notable differences in error handling, particularly with ambiguous terms. For example, in ChemProt (CPR9 → CPR6), Me-LLaMA makes 33.33 % of errors related to lexical overlap for inhibitor terms (e.g., “inhibited”), while LLaMA3 reaches 40 % of errors for similar terms (“inhibitor” and “inhibited”). Additionally, for the CPR4 → CPR9 relation, Me-LLaMA only has 9.52 % errors associated with the term “inhibition”, compared to 33.33 % for LLaMA3. Another example is CPR4 → CPR5, where Me-LLaMA shows 20.69 % errors, while LLaMA3 records 42.42 % errors. For the DDI dataset, regarding the semantic ambiguity between Action and Advise, Me-LLaMA makes 56 % of errors, whereas LLaMA3 makes 60 %. These results suggest that Me-LLaMA, pretrained on domain-specific biomedical corpora, benefits from better exposure to the domain’s particularities, including specialized terms, complex sentence structures, and domain-specific relationships. This allows Me-LLaMA to handle lexical and semantic ambiguities more effectively than LLaMA3. In comparison, T5 committed fewer errors for the ambiguity related to the term “Advise” vs. “Mechanism”, with 39.0 % of errors (Table 8), which is lower than both Me-LLaMA and LLaMA3. This result highlights the effectiveness of T5’s text-to-text architecture, which demonstrates that text generation models are highly adaptable and widely applicable within domain-specific contexts.

### 3.8 Discussion

The role of LLMs in medical practice is an innovative topic that deserves significant attention, especially in real-world applications. Models are a product of the data on which they are trained, and since data collected from real-world scenarios is never perfect due to the inherent limitations of sensing and data collection, computational modeling of real-world systems is inherently limited by the various deficiencies encountered in real data [43]. Even in fields where training datasets can be created, this process remains costly and labor-intensive, particularly in biomedicine, where specialized knowledge is essential [1]. This work explores the use of generative LLMs that can be integrated into the biomedical field without requiring additional training data, and compares their performance with Transformer models fine-tuned for relation extraction. Our results revealed that fine-tuning Transformer models yields higher performance, achieving levels roughly twice those obtained by generative LLMs. This highlights the need for further pretraining on specific data, as demonstrated by Me-LLaMA, which was pretrained on MIMIC-III and shows a notable improvement over LLMs trained exclusively on general-domain data.

In our experiments, we observed notable differences in performance depending on the pretraining corpus used. For instance, T5, which was pretrained only on a general-domain corpus, surprisingly obtained higher F1 scores than BioBERT and PubMedBERT on several biomedical relation extraction tasks. This indicates that model architecture can, in some cases, compensate for the lack of domain-specific data. However, models pretrained on biomedical text generally performed better than those trained solely on general-domain corpora. For example, ClinicalT5, which was trained on clinical notes from MIMIC-III, achieved the highest F1 scores on three out of six biomedical RE datasets compared to T5. Still, we note that PubMedBERT (pretrained on PubMed abstracts) and BioBERT (trained on general and biomedical corpora) did not perform as well compared to T5. On average, the performance gain observed with domain-specific pretraining was +1.96 F-score for PubMedBERT over BioBERT, +4.02 F-score for RoBERTa over BioBERT (RoBERTa was introduced as an improved version of BERT), and +0.5 F-score for ClinicalT5 over T5. Me-LLaMA, pretrained on a biomedical and clinical corpus (MIMIC-III), achieved higher F1 scores than Mistral, LLaMA2, LLaMA3, and Gemma on protein–protein relation extraction. It also outperformed three LLMs on chemical-protein relation extraction, and two LLMs on drug–drug and disease-protein relations. These results highlight that pretraining on domain-specific data has a significant impact on the overall performance.

Thus, LLMs may still require specialized domain knowledge, even after training on diverse datasets, particularly in medical fields where accuracy is crucial. Although LLMs can offer basic guidance, they may not provide precise and reliable suggestions for specific medical issues—especially when extracting complex relationships between biomedical entities. This limitation directly impacts relation extraction tasks, where identifying the correct interaction (e.g., drug-disease, gene-disease) depends on subtle biomedical context. In contrast, domain-specific models leverage structured knowledge and dedicated training to improve precision in relation detection. Knowledge graphs designed for these sectors can offer comprehensive information, ensuring consistent and accurate outputs. Integrating a medical knowledge graph into an LLM could enhance diagnostic accuracy and improve treatment recommendations [44]. Moreover, LLMs must improve their inference capabilities, particularly when handling complex queries involving multiple entities and relationships, such as: “What is the effect of Aspirin on gastrointestinal bleeding in hypertensive patients?”. In such queries, the model must connect multiple layers of information: Aspirin’s antiplatelet action, its impact on bleeding, and the patient’s comorbidities. Current LLMs often fail to infer these multi-hop relations correctly. Knowledge graphs are effective in representing connections and logical thinking, allowing them to perform logical inferences effectively. Therefore, structured data from KGs should be utilized more effectively during LLM inference, instead of leaving everything solely to these structures without further intervention [44]. There are several ways to inject knowledge into LLMs: through prompting (e.g., few-shot learning), via RAG, or by integrating KGs. In this study, we focused on the first two approaches, while future work will explore the integration of KGs with LLMs to provide richer contextual understanding. To address these challenges, future research should focus on optimizing the integration of domain-specific KGs and LLMs by developing scalable, real-time learning models. These models should be capable of dynamically learning from updated KGs, enabling LLMs to adapt to new data and context, especially in relation extraction tasks where entity interactions continually evolve [44]. The combination of KGs and LLMs can significantly enhance clinical decision-making by offering a more detailed and accurate representation of patient data, disease relationships, and treatment options. By leveraging the rich relationships within a KG, LLMs can provide more accurate predictions, assist in diagnosis, and suggest personalized treatment pathways.

## 4 Conclusion

In this paper, we explore fine-tuning Transformer models for biomedical relation extraction tasks, which have demonstrated superior performance. However, this approach requires the creation of domain-specific training data, a process that is both time-consuming and costly due to the need for expert knowledge. As an alternative, generative LLMs offer the advantage of being used directly without extensive data preparation. Despite their convenience, our results show that the performance of generative LLMs remains considerably below that of Transformer models. This highlights the challenges of relying solely on GenAI for real-world biomedical applications and underscores the necessity for additional pretraining of LLMs on clinical data to bridge the performance gap, as demonstrated by Me-LLaMA, pretrained on MIMIC-III, which achieves better results than LLMs trained on general-domain corpora. In addition to domain-specific pretraining, in-context learning (ICL) could provide a promising solution to overcome the high costs of fine-tuning and enable LLMs to learn from biomedical examples. In future work, we plan to explore the integration of knowledge graphs (KGs) as a means to provide structured, domain-specific context that can further enhance pretraining and contribute to the development of more accurate and reliable decision-support systems for real-world biomedical applications.

## CRediT authorship contribution statement

**Hajar El Janah:** Writing – review & editing, Writing – original draft, Visualization, Validation, Software, Methodology, Investigation, Formal analysis, Data curation, Conceptualization. **Youness Nachid-Idrissi:** Writing – review & editing, Resources, Funding acquisition. **Mourad Sarrouti:** Writing – review & editing, Validation, Supervision, Resources, Project administration, Data curation, Conceptualization. **Said Najah:** Writing – review & editing, Validation, Supervision, Resources, Project administration, Funding acquisition, Conceptualization.

## Ethics approval

The Committee for Ethical Guarantees of the Present University conducted a favorable review of the research protocol. Each participant provided informed consent, which elucidated the study’s objectives and the assessment tests employed. Participation was strictly voluntary, with participants retaining the right to withdraw from the study at their discretion. Additionally, all collected data were processed with utmost confidentiality and anonymity.

## Declaration of competing interest

The authors declare that they have no known competing financial interests or personal relationships that could have appeared to influence the work reported in this paper.

## Footnotes

6 Hugging Face, Simple RAG for GitHub issues using Hugging Face Zephyr and LangChain - Open-Source AI Cookbook.

## Footnotes

1 Google, What is generative ai and what are its applications? Google Cloud; 2023. Retrieved February 25, 2024.

2 Niklas Heidloff, Foundation Models, Transformers, BERT and GPT, 2023.

3 Minhajul Hoque, A Comprehensive Overview of Transformer-Based Models: Encoders, Decoders, and More, Medium, 30 April 2023.

4 Hugging Face, BiomedBERT documentation, 2020.

5 Hugging Face, RoBERTa documentation, 2020.
