# Supplementary material for: Exploring transformer models: Fine-tuning VS inference on relation extraction from biomedical texts
Source: Comput Struct Biotechnol J. 2025 Dec 20;31:157–68. doi: 10.1016/j.csbj.2025.12.004 (PMC12796933; doi:10.1016/j.csbj.2025.12.004)
Supplement: Multimedia Component 1 [file mmc1.docx]

| **Exploring Transformer Models: Fine-tuning VS Inference on Relation Extraction from Biomedical Texts** |
| --- |
|  |
| **(Tables)** |

**Table 1.** Statistics of the biomedical RE datasets. For GAD, AIMed and BioInfer, we use the dev set as a test set.

| **Dataset** | **Train** | **Dev** | **Test** | **Metrics** |  |
| --- | --- | --- | --- | --- | --- |
| AIMed | 4938 | 549 | 549 | micro F1 |  |
| BioInfer | 8544 | 950 | 950 | micro F1 | |
| DDI | 2937 | 1004 | 979 | micro F1 |  |
| ChemProt | 4154 | 2416 | 3458 | micro F1 |  |
| DrugProt | 17281 | 3763 | 3765 | micro F1 |  |
| GAD | 4796 | 534 | 534 | micro F1 |  |

**Table 2.** ChemProt relation labels and their associated subgroups [1].

| **Label** | **Subgroups** |
| --- | --- |
| CPR:3 | upregulator, activator, indirect upregulator |
| CPR:4 | downregulator, inhibitor, indirect downregulator |
| CPR:5 | agonist, agonist-activator, agonist-inhibitor |
| CPR:6 | antagonist |
| CPR:9 | substrate, product of |

**Table 3.** Summary of corpora used in BioBERT, based on [2].

| **Corpus** | **Number of words** | **Domain** |
| --- | --- | --- |
| English Wikipedia | 2.5B | General |
| BooksCorpus | 0.8B | General |
| PubMed Abstracts | 4.5B | Biomedical |
| PMC Full-text articles | 13.5B | Biomedical |

**Table 4.** Biomedical relation extraction test results. Models were fine-tuned on six datasets and evaluated on their corresponding validation sets.

| **Relation** | **Dataset** | **BioBERT** | **PubMedBERT** | **T5** | **ClinicalT5** | **RoBERTa** |
| --- | --- | --- | --- | --- | --- | --- |
| Protein–protein | AIMed | 86.52 | 86.89 | 91.80 | **92.90** | 90.34 |
|  | BioInfer | 81.16 | 84.42 | 93.05 | 93.58 | **93.68** |
| Chemical–protein | ChemProt | 90.93 | 93.24 | 92.75 | **93.37** | 90.94 |
|  | DrugProt | 83.98 | 85.07 | 88.41 | **89.74** | 86.45 |
| Drug–drug | DDI | 92.39 | 91.91 | 92.73 | 92.30 | **92.83** |
| Disease–protein | GAD | 71.54 | 76.78 | **80.34** | 80.23 | 76.44 |
| **Average score** |  | 84.42 | 86.38 | 89.85 | **90.35** | 88.44 |

**Table 5.** Biomedical relation extraction test results. Inference models evaluated on four datasets (micro F1-scores with 95% confidence intervals).

**Relation Dataset Mistral-7B LLaMA2-7B GLiNER LLaMA3-8B Gemma RAG Me-LLaMA-13B**

| Protein–protein **AIMed** 28.04 | 19.95  [16.10–23.81] | – | 19.67  [16.58–22.96] | 18.05  [14.76–21.55] | **44.44**  [38.67–51.11] | 38.23  [30.13–46.32] |
| --- | --- | --- | --- | --- | --- | --- |
| Chemical–protein **ChemProt** 45.12 | 34.92 | 34.81 | **76.64** | 55.26 | 55.05 | 52.60 |
| [43.47–46.90] | [33.23–36.69] | [33.14–36.41] | [75.19–78.07] | [53.64–57.04] | [53.28–56.83] | [50.88–54.36] |

[24.35–31.92]

DrugProt

| 32.46  [30.90–33.77] | – | 11.02  [10.09–11.95] | – | – | – | – |
| --- | --- | --- | --- | --- | --- | --- |
| 51.48 | 41.46 | 66.14 | **69.25** | 46.05 | 45.10 | 43.10 |
| [48.31–54.75] | [38.15–44.79] | [62.79–69.64] | [66.39–72.42] | [42.70–49.30] | [41.60–48.61] | [39.75–46.34] |

Drug–drug **DDI**

| Disease–protein | **GAD** | 51.03  [46.90–55.35] | 50.21  [45.76–54.66] | – | | 50.18  [45.88–54.50] | **53.78**  [49.37–58.19] | 44.26  [35.25–53.28] | 49.09  [43.38–55.16] |
| --- | --- | --- | --- | --- | --- | --- | --- | --- | --- |
| **Average score** | 43.92 | | 36.64 | 50.48 | **53.94** | | 43.29 | **47.21** | 45.76 |

*Notes*: GLiNER is deterministic; for LLMs, results were averaged over 10 runs.

**Table 6.** Related Works : Transformers vs Generative LLMs Overview.

| **Related works** | **Datasets** | **RoBERTa** | **BioBERT** | **PubMedBERT** | **T5** | **LLaMA3-8B** | **Me-LLaMA-13B** |
| --- | --- | --- | --- | --- | --- | --- | --- |
|  | **ChemProt** |  | 76.46 [3] |  | 84.82 [3] | 59.03 [4] | 50.52 [4] |
| **our work** | | **90.94** | **90.93** | **93.24** | **92.75** | **76.64** | **52.60** |
| **DDI** | |  |  |  | 82.04 [3] | 65.21 [4] | 76.63 [4] |
| **our work** **92.83** | | | **92.39** | **91.91** | **92.73** | **69.25** | **43.10** |
| **GAD** 80.63 [5] | | | 82.36 [5] | 83.96 [5] |  |  |  |
| **our work** | **76.44** | | **71.54** | **76.78** | **80.34** | **50.18** | **49.09** |
| **Best Score** | 92.83 | | 92.39 | 93.24 | 92.75 | 76.64 | 76.63 |

*Notes:* Comparison of model performance on the ChemProt, DDI, and GAD datasets. The Transformer models were fine-tuned using the same data splits (train, dev, and test) for these datasets. LLaMA3 and Me-LLaMA are fine-tuned variants, whereas our approach relies on inference using these models. F1 scores correspond to micro-F1 values taken from the literature using the same data splits. Our reported scores are highlighted in bold blue. The best scores are indicated in the last row of the table.

**Table 7.** One-shot Prompting for Relation Extraction

**Input:** A set of sentences *S* = {*s*_1_*, s*_2_*, . . . , s_n_*} with entities *e*_1_ and *e*_2_, a language model *f_θ_*, label definitions *L* =

{*l*_1_*, l*_2_*, . . . , l_m_*}, and a prompt with one example per label

**Output:** Predicted relation label *l_i_* for each sentence *s_i_*

1: **for** each sentence *s_i_* in *S* **do**

2: *Prompt Construction:*

3: Prompt = {

4: Task: Identify the relation between *e*_1_ and *e*_2_ in *s_i_*

5: Input: The sentence *s_i_* containing entities *e*_1_ (‘type1’) and *e*_2_ (‘type2’) with their relationship

6: Output: Label from *L*, indicating the relationship between *e*_1_ and *e*_2_

7: Label Definitions: Brief description of each label in *L*

8: One-shot Example: Provide one input–output pair per label to illustrate the task

9: }

10: *Inference:*

11: Send the prompt to the model *f_θ_* to predict the relation label

12: **if** no label is returned **then**

13: Re-send the prompt to regenerate the response

14: **end if**

15: *Postprocessing:*

16: Extract the label from the model output

17: Store the predicted label *l_i_* along with the corresponding sentence *s_i_*

18: **end for**

**Table 8.** EA - The misclassified sentence by the T5 model.

## Examples

1. **AIMed_sentence:** @PROTEIN$ induces chemotaxis and adhesion by interacting with CCR1 and @PROTEIN$. **Gold label: True Predicted label: False**
2. **AIMed_sentence:** A mutant form of human @PROTEIN$ (@PROTEIN$ SC1) that binds one IFN- gamma receptor alpha chain (IFN-gamma R alpha) has been designed and characterized. **Gold label: False Predicted label: True**
3. **BioInfer_sentence:** Integrating the @PROTEIN$ and @PROTEIN$ cytoskeletons. **Gold label:**

## True Predicted label: False

1. **DDI_sentence:** Mixing SYMLIN and Insulin The pharmacokinetic parameters of @DRUG$ were altered when mixed with regular, NPH, and 70/30 premixed formulations of recombinant @DRUG$ immediately prior to injection. **Gold label: mechanism Predicted label: effect**
2. **DDI_sentence:** Due to the chemical similarity of other azole-type antifungal agents (including flu- conazole, @DRUG$, and miconazole) to ketoconazole, and itraconazole, concomitant use of these products with @DRUG$ is not recommended pending full examination of potential interactions.

## Gold label: advise Predicted label: mechanism

1. **ChemProt_sentence:** @CHEMICAL$ and phenformin activate @GENE$ in the heart by increas- ing cytosolic AMP concentration. **Gold label: CPR:3 Predicted label: CPR:5**
2. **ChemProt_sentence:** For some, pharmacologic inhibitors are available, including @CHEMICAL$ for @GENE$, farnesyltransferase inhibitors for NRAS, PD-0325901 for mitogen-activated protein kinase/extracellular signal-regulated kinase kinase, rapamycin analogues for mammalian target of rapamycin, and agents that inhibit either vascular endothelial growth factor or its receptors. **Gold label: CPR:4 Predicted label: CPR:6**
3. **ChemProt_sentence:** Most halogenated @CHEMICAL$ are metabolized by cysteine S-conjugate @GENE$ to pyruvate, ammonia, and an alpha-chloroenethiolate (with DCVC) or an alpha- difluoroalkylthiolate (with TFEC) that may eliminate halide to give a thioacyl halide, which reacts with epsilon-amino groups of lysine residues in proteins. **Gold label: CPR:9 Predicted label:**

**CPR:6**

1. **ChemProt_sentence:** @CHEMICAL$ inhibition of @GENE$. **Gold label: CPR:4 Predicted label: CPR:9**
2. **ChemProt_sentence:** The reaction was inhibited by the specific @CHEMICAL$ inhibitors @GENE$ and fluoxetine. **Gold label: CPR:4 Predicted label: CPR:5**

**Table 9.** Frequency of error types across ChemProt, DDI, and AIMed datasets using T5 model, including the percentage of errors associated with ambiguous terms, the specific terms contributing to each error, and the corresponding error taxonomy.

## Dataset Error Type % Errors with Ambiguous Terms

**% per Term Error Taxonomy**

ChemProt CPR3 → CPR5 57.2% promote 0.8%, increase 23.8%,

activate 13.5%, activator 5.6%,

stimulate 4.0%, reduce 9.5%

**Lexical Overlap:** caused by classes sharing the same words (e.g., CPR3 and CPR5).

ChemProt CPR4 → CPR6 69.6% inhibitors 69.6% **Lexical Overlap:** similar in-

hibitory terms shared between

classes.

ChemProt CPR9 → CPR6 **31.2**% inhibitor 17.0%, inhibited 1.9%,

reduction 1.9%, reduces 1.9%,

are metabolized 8.5%

**Lexical Overlap** for shared in- hibitory terms.

ChemProt CPR4 → CPR9 30.0% inhibition 30.0% **Lexical Overlap**.

ChemProt CPR4 → CPR5 47.2% decrease 6.5%, inhibitor 40.7% **Lexical Overlap:** overlapping

inhibitory and decrease-related

words between classes.

DDI Mechanism → Effect 50.9% Mixing 11.3%, inhibit 18.9%,

metabolism 9.4%, absorption

11.3%

DDI Advise → Mechanism **39.0**% inhibition 12.2%, combination

14.6%, interactions 12.2%

**Semantic Ambiguity: Action vs Result** — terms like "Mix- ing","inhibit" and "metabolism" were often confused as effects rather than mechanisms ;

**Multi-Entity Complexity:** terms like "absorption", created confu- sion by being used in both con- texts of mechanism and effect.

## Semantic Ambiguity: Action vs advise.

AIMed True → False 52.6% "and" 52.6% **Syntactic Ambiguity:** confusion

caused by sentence structure or

conjunctions.

AIMed False → True 71.6% (parentheses) 71.6% **Syntactic Ambiguity:** ambigu-

ous parenthetical phrases.

*Notes*: The arrow (→) indicates misclassification, i.e., the label on the left was misclassified as the label on the right. For example, CPR3 → CPR5 means that CPR3 instances were misclassified as CPR5.

**Table 10.** Frequency of error types across ChemProt, DDI and AIMed datasets using LLaMA3 model, including the percentage of errors associated with ambiguous terms, the specific terms contributing to each error, and the corresponding error taxonomy.

| **Dataset** | **Error Type** | **% Errors with Am- biguous Terms** | **% per Term** | **Error Taxonomy** |
| --- | --- | --- | --- | --- |
| ChemProt | CPR3 → CPR5 | **42.5**% | promote 0%, increase 12.5%,  activate 22.5%, activator 2.5%,  stimulate 5%, reduce 0% | **Lexical Overlap:** caused by classes sharing the same words (e.g., CPR3 and CPR5). |
| ChemProt | CPR4 → CPR6 | **28.89**% | inhibitors 28.89% | **Lexical Overlap:** similar in- hibitory terms shared between classes. |
| ChemProt | CPR9 → CPR6 | 40% | inhibitor 20%, inhibited 20%,  reduction 0%, reduces 0%, are  metabolized 0% | **Lexical Overlap** for shared in- hibitory terms("inhibitor", "in- hibited") |
| ChemProt | CPR4 → CPR9 | 33.33% | inhibition 33.33% | **Lexical Overlap** |
| ChemProt | CPR4 → CPR5 | 42.42% | decrease 0%, inhibitor 42.42% | **Lexical Overlap** confusion due to shared inhibitory term ("inhibitor") |
| DDI | Mechanism → Effect | **25.3**% | Mixing 0%, inhibit 11.7%,  metabolism 0%, absorption  13.6% | **Semantic Ambiguity: Ac- tion vs Result** — term like "inhibit" was often confused as effects rather than mecha- nisms;  **Multi-Entity Complexity:** terms like "absorption", cre- ated confusion by being used in both contexts of mechanism and effect. |
| DDI | Advise → Mechanism | 60% | inhibit 20%, combination 0%,  interactions 40% | **Semantic Ambiguity: Action vs advise**. |
| AIMed | True → False | **0**% | "and" 0% | **Syntactic Ambiguity:** None |
| AIMed | False → True | **50.34**% | (parentheses) 50.34% | **Syntactic Ambiguity:** am- |

biguous parenthetical phrases.

*Notes*: The arrow (→) indicates misclassification, i.e., the label on the left was misclassified as the label on the right. For example, CPR3 → CPR5 means that CPR3 instances were misclassified as CPR5.

**Table 11.** Frequency of error types across ChemProt, DDI, and AIMed datasets using Me-LLaMA model, including the percentage of errors associated with

ambiguous terms, the specific terms contributing to each error, and the corresponding error taxonomy.

| **Dataset** | **Error Type** | **% Errors with Am- biguous Terms** | **% per Term** | **Error Taxonomy** |
| --- | --- | --- | --- | --- |
| ChemProt | CPR3 → CPR5 | 43.75% | promote 0%, increase 12.5%,  activate 6.25%, activator  6.25%, stimulate 12.5%,  reduce 6.25% | **Lexical Overlap:** caused by classes sharing the same words (e.g., CPR3 and CPR5). |
| ChemProt | CPR4 → CPR6 | 57.14% | inhibitors 57.14% | **Lexical Overlap:** similar in- hibitory terms shared between classes. |
| ChemProt | CPR9 → CPR6 | 33.33%  (<% LLaMA3) | inhibitor 0%, inhibited  33.33%, reduction 0%, re-  duces 0%, are metabolized 0% | **Lexical Overlap** for shared in- hibitory terms("inhibited") |
| ChemProt | CPR4 → CPR9 | **9.52**% | inhibition 9.52% | **Lexical Overlap** |
|  |  | (<% LLaMA3) |  |  |
| ChemProt | CPR4 → CPR5 | **20.69**%  (<% LLaMA3) | decrease 0%, inhibitor 20.69% | **Lexical Overlap** confusion due to shared inhibitory term ("inhibitor") |
| DDI | Mechanism → Effect | 31.9% | Mixing 0%, inhibit 17.3%,  metabolism 0%, absorption  14.6% | **Semantic Ambiguity: Ac- tion vs Result** — term like "inhibit" was often confused as effects rather than mecha- nisms;  **Multi-Entity Complexity:** terms like "absorption", cre- ated confusion by being used in both contexts of mechanism and effect. |
| DDI | Advise → Mechanism | 56%  (<% LLaMA3) | inhibit 32.9%, combination  2.4%, interactions 20.7% | **Semantic Ambiguity: Action vs advise**. |
| AIMed | True → False | 20% | "and" 20% | **Syntactic Ambiguity:** confu- sion caused by sentence struc- ture or conjunctions. |
| AIMed | False → True | 53.16% | (parentheses) 53.16% | **Syntactic Ambiguity:** am- |

biguous parenthetical phrases.

*Notes*: The arrow (→) indicates misclassification, i.e., the label on the left was misclassified as the label on the right. For example, CPR3 → CPR5 means that CPR3 instances were misclassified as CPR5.

# References

| [1] | B. V. Team, "Annotation manual of CHEMPROT interactions between CEM and GPRO," 2017. |
| --- | --- |
| [2] | J. L. a. W. Y. a. S. K. a. D. K. a. S. K. a. C. H. S. a. J. Kang, "BioBERT: a pre-trained biomedical language representation model for biomedical text mining," *Bioinformatics,* vol. 36, pp. 1234-1240}, 2019. |
| [3] | L. N. P. a. J. T. A. a. H. T. a. S. C. a. E. B. ˘. G. a. A. P. a. G. Altan-Bonnet, "SciFive: a text-to-text transformer model for biomedical literature," 2021. |
| [4] | M. L. a. H. K. a. H. X. a. R. Zhang, "BiomedRAG: A retrieval augmented large language model for biomedicine," *Journal of Biomedical Informatics,* vol. 162, 2025. |
| [5] | Y. G. a. R. T. a. H. C. a. M. L. a. N. U. a. X. L. a. T. N. a. J. G. a. H. Poon, "Domain-Specific Language Model Pretraining for Biomedical Natural Language Processing}," *ACM Transactions on Computing for Healthcare,* vol. 3, p. 24, 2021. |
