# Supplementary material for: Exploring transformer models: Fine-tuning VS inference on relation extraction from biomedical texts
Source: Comput Struct Biotechnol J. 2025 Dec 20;31:157–68. doi: 10.1016/j.csbj.2025.12.004 (PMC12796933; doi:10.1016/j.csbj.2025.12.004)
Supplement: Multimedia Component 3 [file mmc3.docx]

| **Exploring Transformer Models: Fine-tuning VS Inference on Relation Extraction from Biomedical Texts** |
| --- |
|  |
| **(Supplementary Tables)** |
| Hajar El janah^a^, Youness Nachid-Idrissi^a^ , Mourad Sarrouti^b^, Said Najah^a^ |
|  |
| ^a^ Laboratory of Intelligent Systems and Applications, Faculty of Sciences and Techniques, Sidi Mohamed Ben Abdellah University, Fez, Morocco  ^b^ Sumitovant Biopharma, New York, NY, USA |

**Table A.1** GLiNER - DDI Confusion Matrix.

|  | **Predicted label** | | | |
| --- | --- | --- | --- | --- |
| **Truth label** | **advise** | **effect** | **int** | **mechanism** |
| **advise** | **154** | 14 | 7 | 26 |
| **effect** | 30 | **208** | 16 | 41 |
| **int** | 4 | **25** | **4** | 4 |
| **mechanism** | 14 | 52 | 29 | **146** |

**Table A.2** GLiNER - ChemProt Confusion Matrix.

|  | **Predicted label** | | | | |
| --- | --- | --- | --- | --- | --- |
| **Truth label** | **CPR3** | **CPR4** | **CPR5** | **CPR6** | **CPR9** |
| **CPR3** | **210** | 39 | 121 | 24 | **271** |
| **CPR4** | 412 | **317** | 367 | 59 | **506** |
| **CPR5** | 11 | 2 | **94** | 42 | 46 |
| **CPR6** | 11 | 16 | 48 | **161** | 57 |
| **CPR9** | 108 | 18 | 91 | 5 | **422** |

**Table A.3** Mistral - ChemProt Confusion Matrix.

|  | **Predicted label** | | | | |
| --- | --- | --- | --- | --- | --- |
| **Truth label** | **CPR3** | **CPR4** | **CPR5** | **CPR6** | **CPR9** |
| **CPR3** | **195** | **239** | 74 | 49 | 89 |
| **CPR4** | 216 | **992** | 156 | 99 | 156 |
| **CPR5** | 24 | 46 | **70** | 27 | 25 |
| **CPR6** | 38 | 81 | 37 | **106** | 22 |
| **CPR9** | 95 | 248 | 68 | 48 | **165** |

**Table A.4** Mistral - DDI Confusion Matrix.

|  | **Predicted label** | | | |
| --- | --- | --- | --- | --- |
| **Truth label** | **advise** | **effect** | **int** | **mechanism** |
| **advise** | **96** | 22 | 21 | 82 |
| **effect** | 3 | **125** | 19 | **213** |
| **int** | 1 | 14 | **23** | **58** |
| **mechanism** | 1 | 31 | 10 | **260** |

**Table A.5** LLaMA3 - ChemProt Confusion Matrix.

|  | **Predicted label** | | | | |
| --- | --- | --- | --- | --- | --- |
| **Truth label** | **CPR3** | **CPR4** | **CPR5** | **CPR6** | **CPR9** |
| **CPR3** | **474** | 131 | 40 | 14 | 3 |
| **CPR4** | 89 | **1435** | 33 | 90 | 3 |
| **CPR5** | 4 | 9 | **164** | 17 | 0 |
| **CPR6** | 2 | 3 | 29 | **259** | 0 |
| **CPR9** | 133 | 180 | 12 | 10 | **303** |

**Table A.6** LLaMA3 - DDI Confusion Matrix.

|  | **Predicted label** | | | |
| --- | --- | --- | --- | --- |
| **Truth label** | **advise** | **effect** | **int** | **mechanism** |
| **advise** | **207** | 8 | 1 | 5 |
| **effect** | 33 | **308** | 8 | 11 |
| **int** | 9 | 36 | **42** | 9 |
| **mechanism** | 14 | 154 | 13 | **121** |

**Table A.7** LLaMA3 - GAD Confusion Matrix.

|  | **Predicted label** | |
| --- | --- | --- |
| **Truth label** | **False** | **True** |
| **False** | 22 | **231** |
| **True** | 35 | **246** |
